# Metabolic gene analysis of newly isolated *Pseudochrobactrum* sp. BJQ-1 and BJQ-2 reveals global dissemination of the chlorothalonil hydrolytic dehalogenase *chd* gene

**DOI:** 10.3389/fmicb.2026.1965943

**Published:** 2026-10-06

**Authors:** Zhuang Ke, Qi Wu, Min Gao, Qilin Wang, Lin Wang, Cansheng Yuan

**Affiliations:** 1 College of Rural Revitalization, Jiangsu Open University, Nanjing, China; 2 Key Laboratory of Agricultural and Environmental Microbiology, Ministry of Agriculture and Rural Affairs, College of Life Sciences, Nanjing Agricultural University, Nanjing, China; 3 State Key Laboratory of Microbial Technology, School of Food Science and Pharmaceutical Engineering, Nanjing Normal University, Nanjing, Jiangsu, China

**Keywords:** biodegradation, chlorothalonil, global distribution, horizontal gene transfer, hydrolytic dehalogenase, *Pseudochrobactrum*

## Abstract

Chlorothalonil (TPN; 2,4,5,6-tetrachloroisophthalonitrile) is a globally used, persistent chlorinated fungicide. Its bacterial detoxification is driven mainly by the hydrolytic dehalogenase Chd. Although the *chd* gene and its association with mobile genetic elements have been reported, the extent to which identical *chd* modules have spread across distinct hosts on a global scale remains unclear. Here we isolated two chlorothalonil-degrading strains, BJQ-1 and BJQ-2, from geographically distinct sites. Both were identified as *Pseudochrobactrum* spp. by 16S rRNA phylogeny. Shotgun cloning and sequence comparison revealed that both strains harbor a *chd* ORF essentially identical (≥99.7% nucleotide identity) to those of *Pseudomonas* sp. CTN series, *Paracoccus* sp. XF-3 and *Ochrobactrum lupini* TP-D1. The same *chd* gene is embedded in three distinct mobile-element configurations, an IS*21*-like composite transposon in CTN series, an IS*6*-family transposase TnpA plus Pin recombinase in BJQ-1, and a λ Rac-like invertase in BJQ-2, a pattern incompatible with vertical inheritance. Upstream spacer analysis showed that a conserved IS*21*-like fusion promoter, composed of an IRR-borne −35 box fused to the native −10 element of *chd* gene, and the 179-bp *istB–chd* intergenic spacer (100% identical between the BJQ isolates and the CTN series) are preserved. Mining of the Prokaryotic Genome dataset retrieved *chd*-like sequences from diverse prokaryotic genomes spanning soil, aquatic and plant-associated habitats, confirming a broad phylogeography of the cassette. The recovery of an identical, transposon-associated *chd* module from two distantly sampled *Pseudochrobactrum* strains, together with its global genomic distribution, indicates that the *chd* detoxification cassette has disseminated worldwide, providing a general mechanism for the rapid recruitment of TPN biodegradation capacity in microbial communities.

## 1 Introduction

Chlorothalonil (TPN; 2,4,5,6-tetrachloroisophthalonitrile) is one of the most widely applied broad-spectrum chlorinated fungicides globally. Its environmental persistence, low water solubility, and high toxicity to aquatic organisms, birds, and soil fauna have made TPN residues a critical ecological concern (Van Scoy and Tjeerdema, 2014; Ahmad et al., 2026). Although microbial transformation of TPN proceeds via diverse routes including hydroxylation and cyano hydration in *Stenotrophomonas acidaminiphila* BJ1 (Zhang et al., 2017, 2019) thiolation and nitrile hydration in marine strains (Hu et al., 2020), and cytochrome P450-mediated biotransformation in fungi (Green et al., 2018), hydrolytic dehalogenation remains the dominant transformation pathway. Hydrolytic dehalogenation, catalyzed by the hydrolytic dehalogenase Chd, cleaves an aryl C–Cl bond to yield 4-hydroxychlorothalonil (4-HCTN; Wang et al., 2011; Ren et al., 2011), a transformation that markedly increases the water solubility of the molecule but does not by itself constitute detoxification. Environmental toxicology studies indicate that 4-HCTN is comparably or more toxic and more persistent in aquatic systems than chlorothalonil, and it has been detected in human serum, underscoring its environmental mobility and exposure risk (McMahon et al., 2011; Yang et al., 2025). Complete detoxification of chlorothalonil therefore requires downstream reductive dechlorination of 4-HCTN. A Dehalogenimonas strain designated 4OHTPN1 was recently shown to reductively dechlorinate 4-HCTN via a dedicated reductive dehalogenase gene named *htpnA*, extending the known TPN catabolic network beyond the initial Chd step (Liu et al., 2026).

The known host range of chd has expanded considerably since its initial cloning from *Pseudomonas* sp. CTN-3 (Wang et al., 2010). The CTN series comprising CTN-1 to CTN-16 was isolated from multiple TPN-contaminated sites in China and includes 16 strains across eight genera including *Ochrobactrum, Shinella, Caulobacter, Rhizobium, Bordetella, Pseudoxanthomonas, Pseudomonas*, and *Lysobacter* (Liang et al., 2011). Identical *chd* cassettes have also been identified in *Rhodococcus* sp. XF-6 and XF-8 (Liang et al., 2011, 2012), *Ochrobactrum lupini* TP-D1 (Ren et al., 2011), *Paracoccus* sp. XF-3 (Yue et al., 2015), *Pseudochrobactrum* sp. BSQ-1 (Liu et al., 2016), and the two *Pseudochrobactrum* isolates described herein. Field isolates designated RB-4, RB-5, RB-9, RB-16, RB-28, RB-29, and RB-38 from a long-term TPN-contaminated site also carry *chd* gene linked to the IS-*Olup* transposon (Ren et al., 2011). Notably, *chd* is not restricted to a single phylogenetic lineage. In *O. lupini* TP-D1 the gene resides on an IS*21*-like composite transposon named IS-*Ocsp1* which harbors *istA, istB*, and *chd*. An upstream insertion element designated IS-*Olup* recurs in seven additional degradative strains spanning *Pseudomonas, Achromobacter, Ochrobactrum, Ralstonia*, and *Lysobacter* (Ren et al., 2011). Liang et al. (2011) further demonstrated that 16 TPN-dechlorinating strains from eight genera all carry *chd* gene in close association with IS-*Ocsp1* and suggested that horizontal gene transfer facilitates rapid bacterial adaptation to TPN pollution. This mobility of dehalogenase genes has since been framed as a general mechanism for microbial community adaptation in chlorinated-compound-contaminated ecosystems (Liang et al., 2011). Mobile genetic elements are more broadly recognized as the primary drivers for the assembly and dissemination of xenobiotic catabolic pathways because they enable beneficial genes to emerge abruptly in phylogenetically distant hosts at contaminated sites (Top and Springael, 2003; Phale et al., 2019).

Despite these advances, two aspects of *chd* dissemination remain underexplored. Whether distinct strains from distant sites carry identical rather than merely homologous *chd* modules, including the flanking mobility genes, is still unresolved. Nucleotide-level identity is the strongest field-level evidence for recent horizontal transfer, because independent recruitment would not preserve the surrounding sequence. The full taxonomic and habitat breadth of *chd* dissemination is equally unclear and can only be addressed by database-wide mining rather than reliance on cultured isolates. In this study we isolated two chlorothalonil-degrading strains, BJQ-1 and BJQ-2, from two geographically separated locations and identified both as *Pseudochrobactrum* by 16S rRNA phylogeny. We show that both carry a *chd* module essentially identical to the CTN/XF/TP-D1 chd, including associated transposase genes, and that *chd* gene is embedded in at least three distinct mobile-element configurations among reported carriers. We further demonstrate that an IS*21*-like fusion promoter, an IRR-borne −35 box fused to the native −10 element of *chd* gene, is conserved between the CTN series and the BJQ isolates, and that mining of the Prokaryotic Genome dataset recovers *chd*-like sequences from diverse genomes and habitats. Collectively, the data indicate that a pre-activated, mobile *chd* transformation cassette has spread globally.

## 2 Materials and methods

### 2.1 Chemicals, bacterial strains, plasmids, primers, and culture conditions

The standard compounds of chlorothalonil (TPN) and 4-hydroxychlorothalonil (4-HCTN) were purchased from J&K Scientific Ltd. (Shanghai, China) with purities of ≥98%. High-performance liquid chromatography (HPLC)-grade methanol and acetonitrile were purchased from Merck Co., Ltd. (Darmstadt, Germany). All other reagents used in this study were of analytical grade. Bacterial strains and plasmids used in this study are described in Table 1. Luria–Bertani (LB) medium (10.0 g tryptone, 5.0 g yeast extract and 5.0 g NaCl per liter of water, pH 7.0) was used to culture *Escherichia coli* and BJQ strains aerobically at 30 °C. Mineral salts medium (MSM; 1.0 g NH_4_Cl, 1.5 g K_2_HPO_4_, 0.5 g KH_2_PO_4_, 0.2 g MgSO_4_, and 1.0 g NaCl per liter of water, pH 7.0) was used for strain isolation and degradation assays. The solid medium was prepared by adding 2.0% (w/v) agar. Antibiotics were added as necessary at the following concentrations: ampicillin (Amp), 100 mg L^−1^; kanamycin (Km), 50 mg L^−1^.

**Table 1:** Bacterial strains and plasmids used in this study.

Strains and plasmids	Relevant characteristics	Source or reference
BJQ-1	TPN-degrading strain, wild type	This study (Huaian farmland soil)
BJQ-2	TPN-degrading strain, wild type	This study (Xinyi abandoned pesticide factory)
*E. coli* DH5α	Host strain for cloning	Lab stored
*E. coli* DH10B	Host strain for genomic library	Lab stored
*E. coli* BL21(DE3)	Host strain for expression	Lab stored
pET-28a(+)	Expression vector, Km^r^	Lab stored
pUC-18	Shotgun-cloning vector for genomic library, Amp^r^	Lab stored

Amp^r^, ampicillin resistance; Km^r^, kanamycin resistance.

### 2.2 Strain isolation, genome sequencing and 16S-based identification

Soil samples were collected from chlorothalonil-treated farmland in Huaian, Jiangsu Province, China (BJQ-1) and an abandoned pesticide factory in Xinyi, Xuzhou, Jiangsu Province (BJQ-2). TPN-degrading strains were isolated via continuous enrichment as described by Yue et al. (2015). Briefly, 10 g of each soil was incubated in 90 mL minimal salt medium (MSM) with 0.2 mM TPN at 30 °C and 150 rpm; 5 mL aliquots were transferred to fresh medium every 5 days for five cycles. Diluted cultures were then plated on MSM agar containing 0.2 mM TPN, and colonies exhibiting clear halos after 5 days at 30 °C were purified and designated BJQ-1 and BJQ-2.

Genomic DNA was extracted using a Bacterial DNA Kit (Omega Bio-Tek), and genomes were sequenced on an Illumina NovaSeq 6000 platform by Biozeron Biotechnology (Shanghai, China). Gene annotation was performed against UniProtKB/Swiss-Prot, NR, KEGG, and COG databases via BLAST, supplemented by RAST analysis. Average nucleotide identity (ANI) and digital DNA–DNA hybridization (dDDH) were calculated using OrthoANIu (www.ezbiocloud.net/tools/ani) and the GGDC server (Meier-Kolthoff et al., 2013), respectively. Full-length 16S rRNA genes retrieved from genome sequences were compared against EzBioCloud and GenBank type-strain databases. Phylogenetic trees for both strain identification and broader host-range analysis (including 24 *chd*-carrying strains) were constructed using maximum-likelihood inference in IQ-TREE under the Kimura 2-parameter model with 1,000 bootstrap replicates.

### 2.3 Chlorothalonil degradation assays

Cells of strains BJQ-1 and BJQ-2 were cultured in LB broth at 30 °C and 160 rpm to mid-logarithmic phase, harvested by centrifugation (6,000 × g, 5 min), and washed twice with sterile MSM. The cells were resuspended and inoculated into 100 mL MSM containing 0.42 mM TPN at an initial OD_600_ of 0.2, then incubated at 30 °C with shaking at 160 rpm. Heat-killed cells served as abiotic controls. At 3-h intervals, 3-mL aliquots were withdrawn, centrifuged (12,000 × g, 5 min, 4 °C), and analyzed for residual TPN by HPLC as described above.

The effects of temperature, pH, and metal ions on TPN degradation were evaluated. Temperature gradients (15, 20, 25, 30, 37, 40, and 45 °C) were tested at 0.2 mM TPN and pH 7.0; pH gradients (3.0, 4.0, 5.0, 6.0, 7.0, 8.0, 9.0, and 10.0) were assessed at 30 °C and 0.2 mM TPN; and individual metal ions (Mn^2+^, Zn^2+^, Mg^2+^, Ca^2+^, Cu^2+^, Fe^3+^, Ni^2+^, Pb^2+^, Co^2+^, Al^3+^) were tested at 0.1 mM under standard conditions (30 °C, pH 7.0, 0.2 mM TPN). All experiments were performed in triplicate.

### 2.4 Molecular cloning, expression and characterization of *chd*

Genomic libraries of BJQ-1 and BJQ-2 were constructed in *E. coli* DH10B using pUC18 with partial *Sau3*AI-digested inserts and screened for TPN-transforming activity on solid medium containing TPN. Positive clones were isolated and sequenced by Tsingke Biotechnology (Beijing, China). The *chd* gene was amplified from genomic DNA using primers chd-F (5′-gtcgacggagctcgaattctcaaggcctggctgcgag-3′, EcoR I) and chd-R (5′-caaatgggtcgcggatccatgccactcaagttttcgggc-3′, BamH I), cloned into pET-28a(+), and transformed into *E. coli* BL21(DE3) for expression. Transformants were cultured at 37 °C in LB broth supplemented with 50 mg L?^1^ kanamycin to an OD_600_ of 0.6, induced with 0.4 mM IPTG, and incubated at 16 °C for 20 h with shaking at 160 rpm. Cells were harvested, resuspended in lysis buffer (50 mM Tris-HCl, 300 mM NaCl, pH 8.0), and disrupted by sonication. After centrifugation at 16,000 × g for 15 min at 4 °C, the supernatant was loaded onto a cobalt-affinity column and eluted with imidazole gradients (10–200 mM) in the same buffer. Purified Chd was verified by SDS-PAGE and dialyzed against 50 mM Tris-HCl (pH 8.0). Enzymatic activity was confirmed by monitoring TPN degradation and 4-HCTN formation via HPLC as described in Analytical methods part, and by spotting purified protein onto LB plates containing 0.2 mM TPN in 50 mM Tris-HCl (pH 8.0) to visualize hydrolysis zone and confirm enzyme activity.

### 2.5 Comparative *chd* cluster and promoter structure

Genome assemblies for BJQ-1, BJQ-2, and 13 additional *chd*-harboring strains were retrieved from GenBank or generated through in-house sequencing. The reference strains included *Pseudomonas* sp. CTN-3, *O. lupini* TP-D1, *Paracoccus* sp. XF-3, *Rhodococcus* spp. XF-6 and XF-8, *Niastella koreensis* GR20-10 (Weon et al., 2006), and CTN-1, CTN-8, CTN-10, CTN-11, CTN-13, CTN-14, and CTN-15. Coding sequences in each assembly were predicted using Prokka (Seemann, 2014) and compared against the BJQ-1 proteome and genomic sequence via BLASTP and BLASTN, respectively. A coding sequence was retained as a *chd* cluster homolog when its best hit satisfied ≥30% identity, an *E*-value ≤ 1 × 10^−3^, and ≥50% query coverage. Each locus was extracted from the nearest upstream mobility gene to the first downstream gene relative to chd. All loci were oriented with *chd* reading 5′ to 3′ and plotted on a shared kilobase axis with per-gene amino acid identity to BJQ-1 Chd indicated.

For the *chd* upstream region, the intergenic sequence between the terminal mobility gene (*istB* or its equivalent) and the *chd* start codon was extracted together with 40 bp of the *chd* leader, oriented to the *chd* strand, and aligned globally to the BJQ-1 spacer with MAFFT L-INS-i (Katoh and Standley, 2013). This 179-bp segment (intergenic region plus 40 bp of leader) defines the *istB*–*chd* intergenic spacer. Inverted repeats and promoter motifs were identified by ungapped sliding-window scanning of the BJQ-1 spacer against the consensus sequences reported by Liang et al. (2011), namely IRL (TGTTGCGCGCAGAGTAAAACTGGGCCACTTCGCGC), IRR (GCGCGAAGTGGCTCAGATTTGGGCTGCGCTTGACA), the direct repeat TGGG, and an outward −35 box of the TTGACA family embedded in the IRR. Pairwise identity of each spacer to BJQ-1 was derived from the global alignment.

### 2.6 Global mining of *chd* homologs and phylogenetic analysis

To assess the global distribution of *chd*, predicted proteins from the Prokaryotic Genome dataset, which comprises 200,532 genomes compiled from literature and 157,560 genomes from Integrated Microbial Genomes & Microbiomes (Chen et al., 2021; Mukherjee et al., 2021), were aligned against the Chd amino acid sequence of strain BJQ-1 using DIAMOND v2 (Buchfink et al., 2021). The search parameters were set to ≥50% identity, >50% query coverage, and an *E*-value < 1 × 10^−10^ to capture diverse *chd*-like homologs. This screening retrieved 104 homologs from 104 distinct genomes. Genome taxonomy was assigned with GTDB-Tk v2 (Chaumeil et al., 2022; Parks et al., 2020) and a maximum-likelihood tree of the homologous proteins was built with FastTree v2 (Price et al., 2010).

For gene-level phylogenetic analysis, the 104 mined *chd*-like amino acid sequences were combined with five previously reported Chd protein sequences from known chlorothalonil-degrading strains that lack publicly available genome assemblies. This *chd-*like homologs sequences were aligned with MAFFT L-INS-i (Katoh and Standley, 2013), trimmed with trimAl (Capella-Gutiérrez et al., 2009), and inferred with IQ-TREE v2 (Minh et al., 2020; –m Q.pfam+R5, 1,000 ultrafast bootstrap replicates). Trees were visualized with iTOL (Letunic and Bork, 2021). Habitat assignment of retrieved genomes followed the corresponding genome metadata (isolation source / biome).

### 2.7 Analytical methods

HPLC analysis was performed using a Dionex UltiMate 3000SD system equipped with a diode array detector to qualitatively and quantitatively determine TPN and 4-HCTN. For TPN quantification, separation was achieved on a Thermo C_18_ reversed-phase column (4.6 × 250 mm, 5 μm particle size) maintained at 30 °C. The mobile phase consisted of methanol and ultrapure water (90:10, v/v) delivered at a flow rate of 1 mL min^−1^. The injection volume was 20 μL, and detection wavelengths were set at 232 and 260 nm. Under these conditions, authentic TPN exhibited a retention time of 3.17 min. For 4-HCTN quantification, a Thermo Hypersil GOLD PFP column (4.6 × 250 mm, 5 μm particle size) was employed at 40 °C. The mobile phase comprised acetonitrile and 0.1% aqueous phosphoric acid (65:35, v/v) at a flow rate of 1 mL min^−1^, with an injection volume of 20 μL and detection at 221 and 241 nm. Authentic 4-HCTN showed a retention time of 13.38 min under these conditions.

- 4-HCTN was identified by LC-QTOF-MS/MS following Wang et al. (2026). Culture supernatants at peak 4-HCTN accumulation were extracted with dichloromethane, dried under nitrogen, reconstituted in methanol to 1 mL, and filtered through a 0.22-μm membrane. Analysis was performed on an Agilent 1260 Infinity HPLC coupled to a TripleTOF 5600 mass spectrometer (AB Sciex) using an XBridge BEH C^18^ column (3.5 μm, 2.1 × 100 mm; Waters) at 20 °C. Isocratic elution with water and methanol (60:40, v/v) was delivered at 200 μL min^−1^. The mass spectrometer operated in negative ESI mode scanning m/z 30–500 with ionspray voltage −4,500 V, source temperature 550 °C, gas pressures 55 psi, curtain gas 35 psi, declustering potential −80 V, and collision energy −10 V. MS/MS acquisition used collision energies of 25–45 V with nitrogen as the nebulizing, drying, and curtain gas. Data were calibrated externally to within 3 ppm mass accuracy and processed with Analyst TF 1.6 and PeakView 1.2 (AB Sciex). 4-HCTN was confirmed by exact mass and diagnostic fragments, with observed [M–H]^−^ at m/z 244.9079 for BJQ-1 and 244.9080 for BJQ-2 matching the theoretical value of 244.9076.

### 2.8 Nucleotide sequence accession numbers

The full-length 16S rRNA gene sequences of BJQ-1 and BJQ-2 were deposited in GenBank under accession PZ225271 and PZ225286, respectively. The nucleotide sequences containing the *chd* gene together with its upstream and downstream flanking regions, i.e., the cloned *chd* cassettes of BJQ-1 and BJQ-2, were also deposited in GenBank under accession PZ786007 and PZ786008, respectively.

### 2.9 Statistical analysis

All experiments were performed in triplicate. Data are presented as the mean ± standard deviation (SD). Significant differences among treatments were assessed by one-way ANOVA followed by Tukey's HSD *post-hoc* test (*P* < 0.05) using GraphPad Prism (version 9). Different lowercase letters above bars denote statistically significant differences (*P* < 0.05).

## 3 Results

### 3.1 Molecular characterization of TPN degradation by BJQ-1 and BJQ-2

Two chlorothalonil-degrading strains designated BJQ-1 and BJQ-2 were isolated from a chlorothalonil-treated farmland in Huaian and an abandoned pesticide factory in Xinyi, Xuzhou, respectively. Both isolates produced clear halos on TPN-supplemented agar to indicate their capacity for extracellular TPN degradation (Supplementary Figure S1). Resting-cell assays performed with 0.42 mM TPN at an initial OD_600_ of 0.2 and 30 °C demonstrated that both strains converted TPN to 4-HCTN without a detectable lag phase. BJQ-1 completely eliminated the substrate within 12 h while BJQ-2 achieved complete degradation within 15 h. The metabolite 4-HCTN accounted for approximately 97% of the consumed TPN across both strains. No TPN loss or product formation was observed in abiotic controls, confirming that the transformation was biologically mediated (Figure 1).

**Figure 1:**
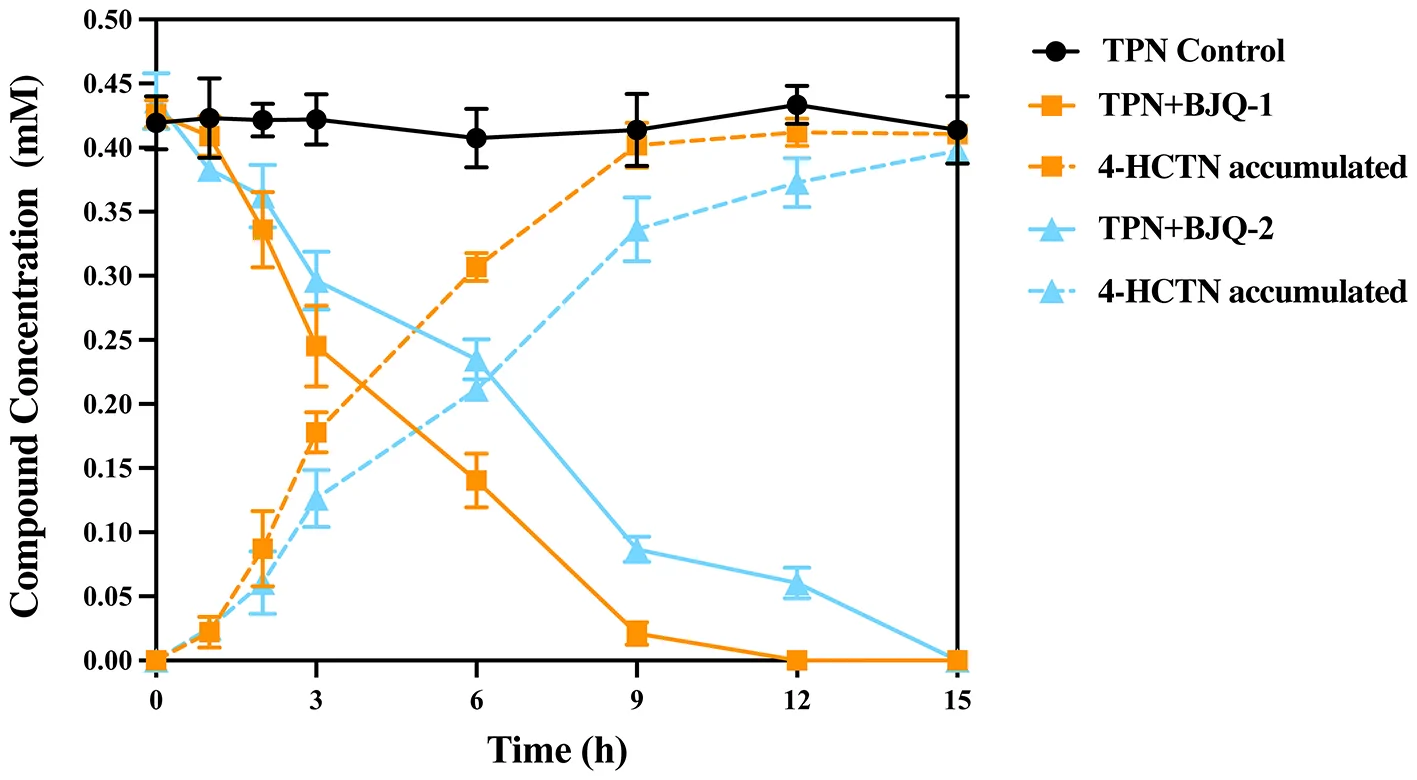
Degradation of chlorothalonil by strains BJQ-1 and BJQ-2 as determined by resting-cell assays. The time-course was measured in MSM containing 0.42 mM TPN at an initial OD_600_ of 0.2 and 30 °C. Solid and dashed lines indicate chlorothalonil removal and TPN-OH accumulation, respectively; orange and light blue denote the addition of strains BJQ-1 and BJQ-2, respectively.

Environmental parameters significantly affected TPN degradation activity in both isolates. Both strains exhibited similar temperature profiles with peak activity at 30 °C and a sharp decline at 45 °C to indicate a narrow thermal tolerance range despite their distinct geographic origins. The two isolates showed an essentially identical pH response, both reaching maximum activity near pH 7.0 with a secondary peak at pH 8.0. Activity persisted at approximately 60% of maximum at pH 4.0 but dropped to about 40% at pH 9.0, indicating that both strains remain substantially active across acidic to alkaline conditions. Metal ion screening revealed distinct effects among the 10 ions tested. Mg^2+^, Zn^2+^, and Co^2+^ maintained or slightly enhanced TPN removal whereas Cu^2+^, Mn^2+^, and Ni^2+^ strongly inhibited degradation to suggest a metal-sensitive step in the dehalogenation reaction (Supplementary Figure S2).

### 3.2 Chd-mediated TPN dehalogenation *in vitro*

Shotgun cloning of partially *Sau3*AI-digested genomic DNA into *E. coli* DH10B yielded colonies that formed clear halos on TPN-supplemented plates (Supplementary Figure S3). Sequencing of the positive inserts identified a single chd open reading frame in each strain. These genes shared ≥99.7% nucleotide identity with known chd genes from the CTN series, *Paracoccus* sp. XF-3 and *Ochrobactrum lupini* TP-D1. The Chd proteins were expressed in *E. coli* BL21(DE3) using pET-28a(+) and purified via cobalt-affinity chromatography to yield a single soluble band of approximately 33 kDa (Supplementary Figure S4). The purified enzymes converted TPN to 4-HCTN *in vitro* and the product co-eluted with the metabolite produced by the parental strains. LC–QTOF-MS/MS analysis in negative ion mode detected [M–H]^−^ ions at m/z 244.9079 for BJQ-1 and 244.9080 for BJQ-2. These values matched the theoretical mass of 4-HCTN (m/z 244.9076) within 3 ppm. Diagnostic fragment ions at m/z 209.94, 181.95, 174.97, 146.98, and 112.01 further confirmed the product identity (Figure 2). Purified Chd also produced clear hydrolysis zones when spotted onto LB plates containing 0.2 mM TPN to demonstrate that the cloned gene alone is sufficient for the hydrolytic dehalogenation observed *in vivo* (Supplementary Figure S5).

**Figure 2:**
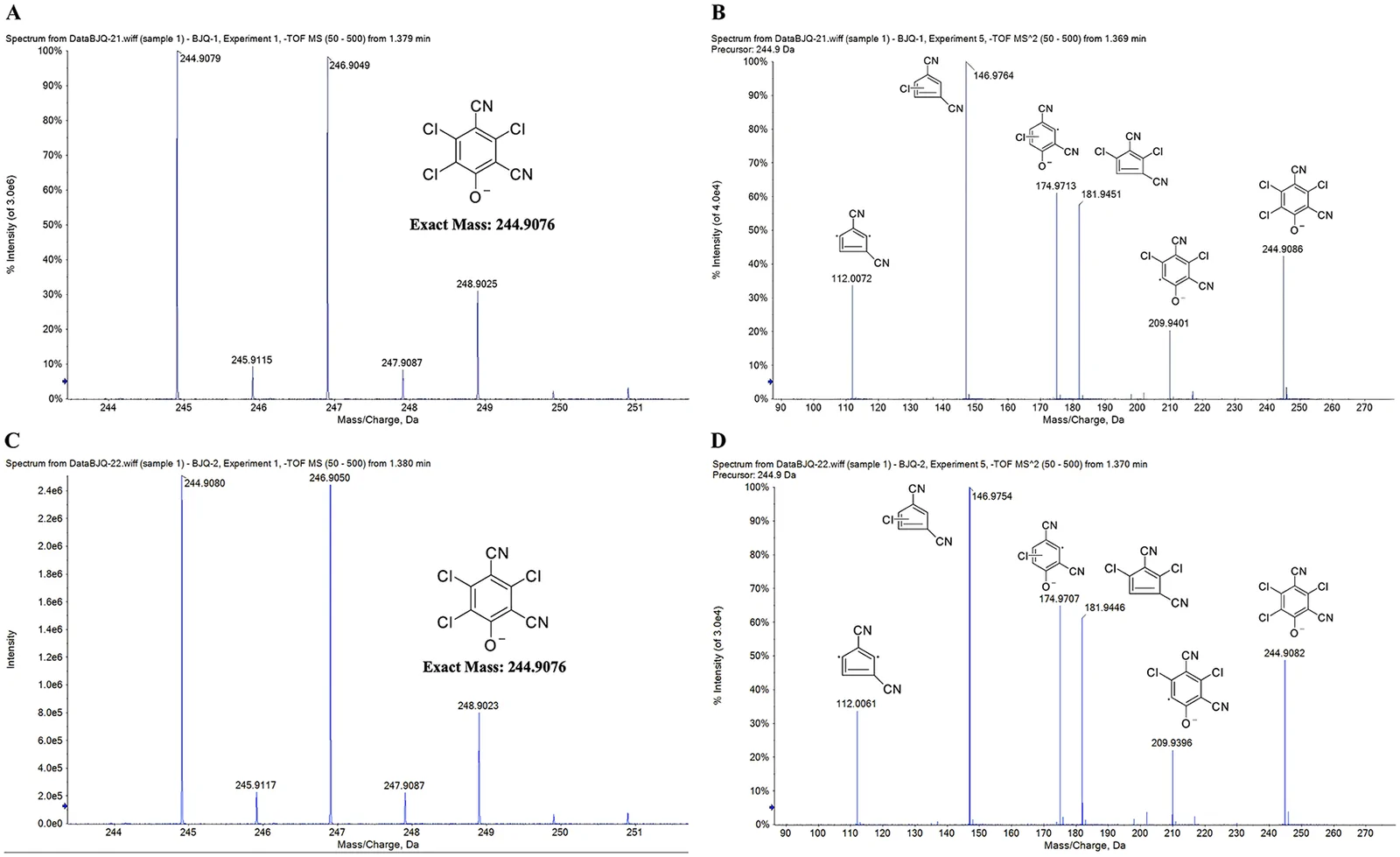
LC–MS identification of chlorothalonil metabolites. High-resolution TOF-MS spectrum **(A, C)** and MS/MS spectrum **(B, D)** of the metabolite 4-HCTN generated by strains BJQ-1 and BJQ-2, respectively. The deprotonated molecule [M–H]^−^ was observed at m/z 244.9079 (BJQ-1) and 244.9086 (BJQ-2), in close agreement with the theoretical value of 244.9076 Da (error < 0.4 ppm). Key diagnostic fragment ions at m/z 209.9401, 181.9451, 174.9713, 146.9764, and 112.0072 support the structural identity of 4-HCTN.

### 3.3 Taxonomic position of BJQ-1 and BJQ-2 and distribution of chd among bacterial taxa

Full-length 16S rRNA gene sequences recovered from the genome assemblies were analyzed alongside those of *Pseudochrobactrum* type strains and related alphaproteobacterial reference taxa. In the maximum-likelihood tree, both isolates clustered within the genus *Pseudochrobactrum* and formed a strongly supported clade with *Pseudochrobactrum* sp. BSQ-1 (Figure 3), a chlorothalonil degrader previously isolated from a different site. BJQ-1 showed 99.93% sequence similarity to *P. saccharolyticum* CCUG 33852 and 99.86% to the type species *P. asaccharolyticum* DSM 25619. BJQ-2 was most closely related to *P. asaccharolyticum* DSM 25619 at 99.64% similarity. Although these values confirm generic assignment, 16S rRNA identity above 99% lacks the resolution required for species delineation. Whole-genome comparisons were therefore performed against the type strain *P. asaccharolyticum* DSM 25619. BJQ-1 and BJQ-2 shared an average nucleotide identity (ANI) of 86.1% and a digital DNA–DNA hybridization (dDDH) value of 29.9%. Against DSM 25619, BJQ-1 exhibited an ANI of 85.9% and dDDH of 29.7%, while BJQ-2 showed an ANI of 94.95% and dDDH of 59.4%. All pairwise ANI and dDDH values remained below the accepted prokaryotic species thresholds of 95% and 70%, respectively (Jain et al., 2018; Goris et al., 2007). Although the BJQ-2 vs. DSM 25619 comparison approached the ANI threshold, it did not meet the criteria for conspecificity. Consequently, BJQ-1 and BJQ-2 represent two distinct novel species within*Pseudochrobactrum*.

**Figure 3:**
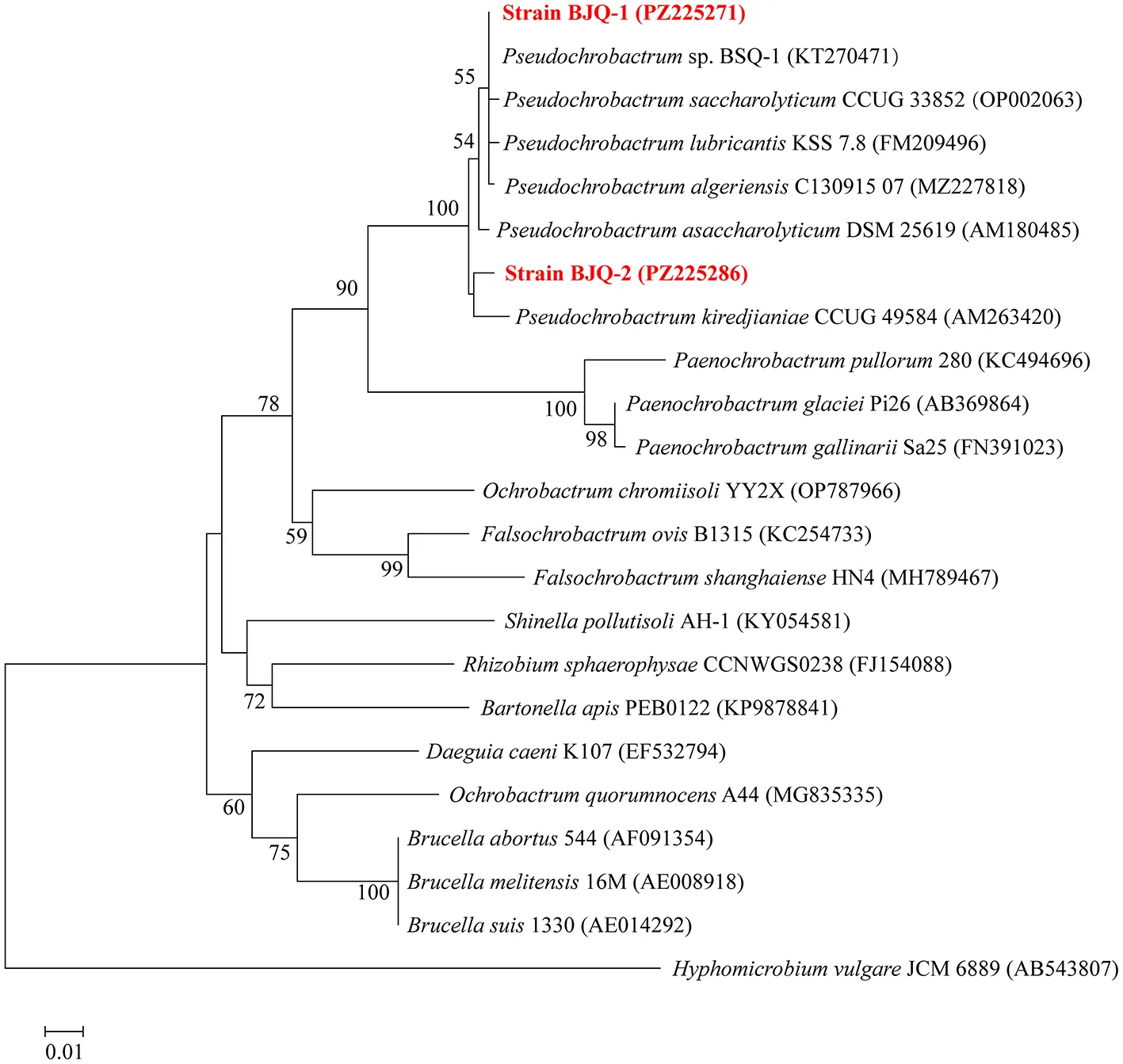
Phylogenetic analysis of strains BJQ-1 and BJQ-2 with closely related type strains based on 16S rRNA gene sequences using the maximum-likelihood method. Bootstrap values (%) are indicated at the nodes, and the scale bar represents 0.01 substitutions per site. GenBank accession numbers are shown in parentheses.

A broader 16S rRNA phylogeny comprising 26 *chd*-carrying strains revealed that the gene is distributed across five bacterial classes (Figure 4). These hosts include Gammaproteobacteria (*Pseudomonas, Pseudoxanthomonas*, and *Lysobacter*), Betaproteobacteria (*Bordetella*), Actinobacteria (*Rhodococcus*), Alphaproteobacteria (*Caulobacter, Paracoccus, Ochrobactrum, Shinella, Rhizobium*, and *Pseudochrobactrum*, including BJQ-1 and BJQ-2), and Bacteroidia (*Niastella koreensis* GR20-10). The BJQ isolates clustered within the Alphaproteobacteria and were phylogenetically distant from both the gammaproteobacterial CTN series and the actinobacterial *Rhodococcus* strains. The presence of nearly identical detoxification genes across such broad taxonomic distances argues against vertical inheritance from a common ancestor.

**Figure 4:**
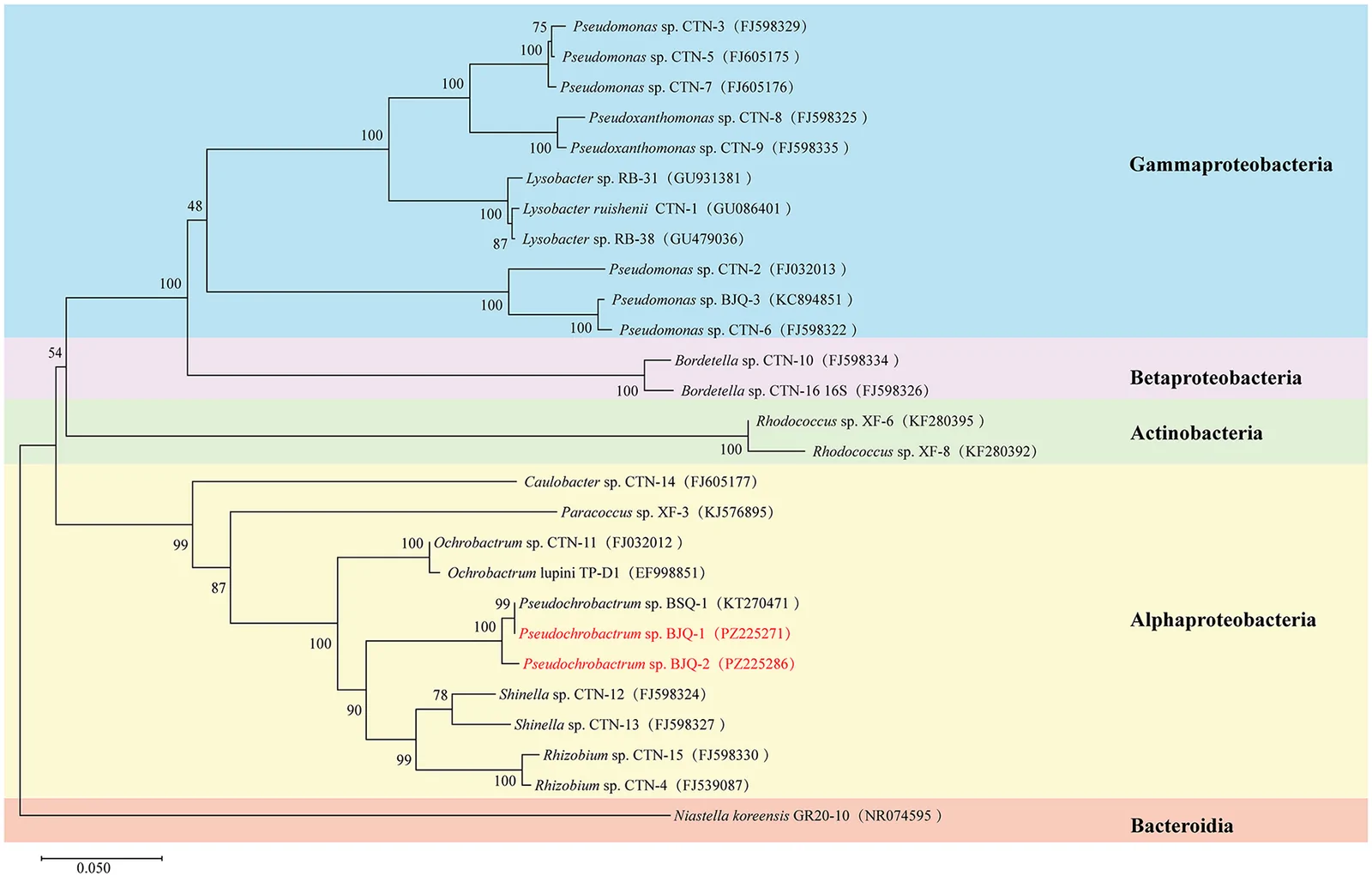
16S rRNA gene phylogeny of 26 *chd*-carrying strains. The tree comprises 24 reported or NCBI-retrieved strains, along with BJQ-1 and BJQ-2 from this study. Phylogenetic inference was performed using the maximum-likelihood method in IQ-TREE under the Kimura 2-parameter model with 1,000 bootstrap replicates.

### 3.4 Conservation and divergence of the chd cassette in three mobility configurations

Comparative analysis of 15 strains revealed three distinct mobile-element configurations surrounding the *chd* gene (Figure 5). In the CTN series, *chd* was flanked by the IS*21*-like composite transposon IS-*Ocsp1* with *istA* and *istB* located immediately upstream. CTN-11 also carried a mercury-resistance module (*merE, merD*, and *merB*) adjacent to the cassette. The *chd* loci of BJQ-1 and BJQ-2 are located on chromosomal contigs. In BJQ-1, *chd* was associated with an IS*6*-family transposase (*tnpA*) and a Pin recombinase while retaining an *istB*-like gene directly upstream. In BJQ-2, *chd* gene was preceded by a λ Rac-like invertase alongside an *istB*-like gene. Despite this structural divergence, the Chd protein sequence was 99.4–100% identical across the 14 core carriers. The *N. koreensis* GR20-10 Chd-like sequence with 44.5% identity served as an outgroup. The flanking mobility genes were significantly less conserved. The *istB* gene shared 95.7–100% identity among IS-*Ocsp1* strains but *istA* and the IS*6-tnpA* in BJQ-1 showed only 55.6% identity to their CTN counterparts. Upstream of chd, the 179-bp *istB*–*chd* intergenic spacer retained the IS*21*-like fusion-promoter architecture across all configurations. This architecture comprises the IRR, embedded TTGACA −35 box and downstream TGGG direct repeat (Supplementary Figure S6). This spacer was 100% identical among BJQ-1, BJQ-2, and CTN-1. It was 99.4% identical in CTN-8/13/14, 79.5% identical in CTN-10/15 and 67.5% identical in CTN-11 where it was interrupted by the mer module. The high conservation of this spacer across different genera suggests that dissemination occurred on a recent evolutionary timescale. The near-invariance of *chd* coupled with the divergence of flanking mobile elements into three distinct configurations indicates that the enzyme and its carriers do not share a single evolutionary history. This pattern is consistent with recurrent and independent recruitment of the same detoxification gene by different mobile elements rather than vertical inheritance via a single conserved transposon.

**Figure 5:**
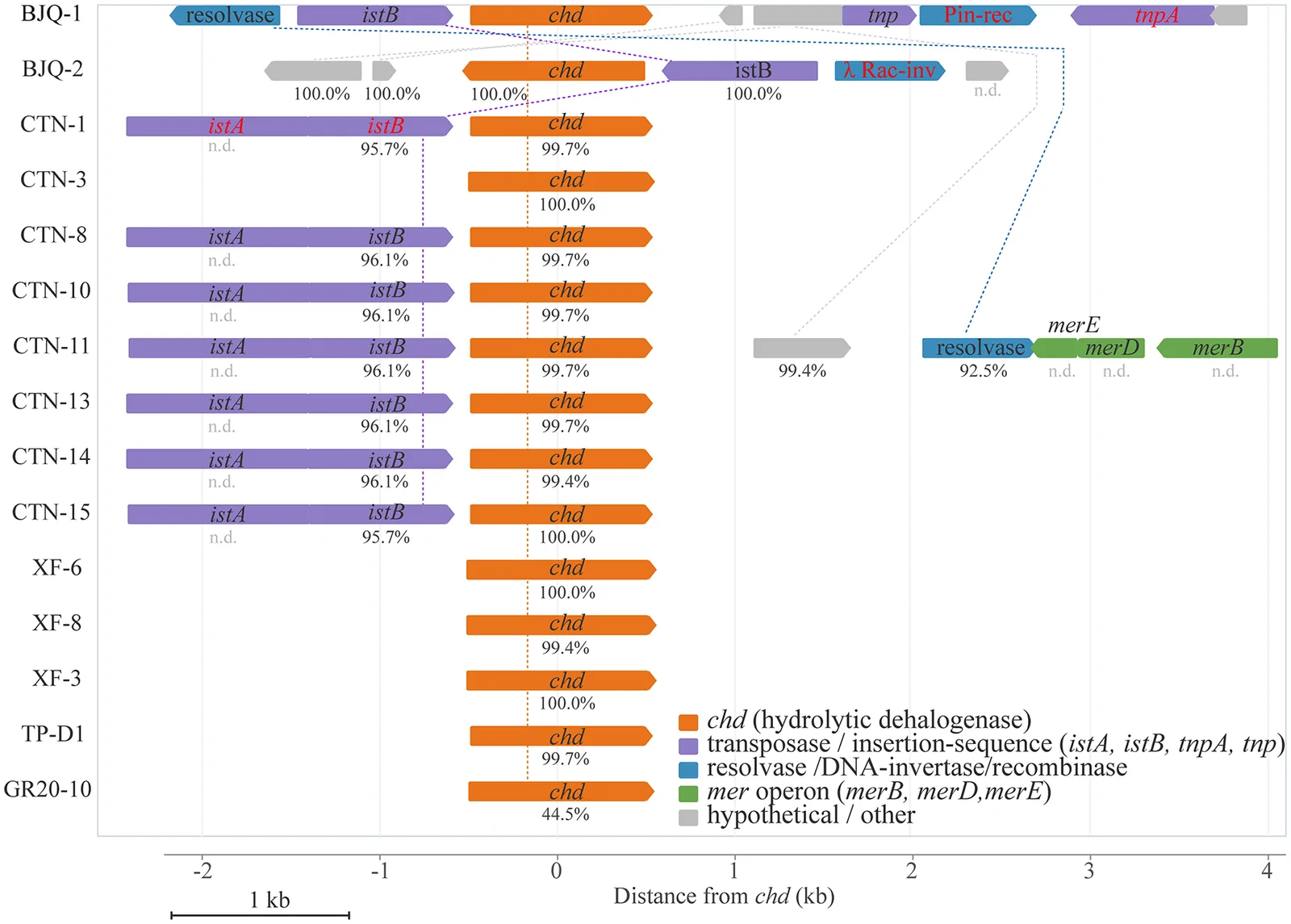
Comparative analysis of *chd*-centered multigene clusters across 15 *chd*-positive strains. Gene tracks are drawn to scale on a shared kilobase (kb) axis. Color coding indicates *chd* (orange), IS/mobility genes (purple), resolvase/Pin (blue), *mer* cluster (green), and hypothetical proteins (gray). Percent identity of each CDS to the Chd protein of BJQ-1 is indicated above the corresponding gene, with BJQ-1 serving as the top reference track. Three distinct mobility configurations are highlighted, including IS21 (CTN), IS6 + Pin (BJQ-1), and λ Rac invertase (BJQ-2).

### 3.5 Global phylogeography of chd homologs

Screening the Prokaryotic Genome dataset (358,092 genomes) with DIAMOND (identity ≥50%, query coverage >50%, *E*-value < 1 × 10^−10^) retrieved 104 *chd*-like sequences from distinct genomes, corresponding to a hit rate of approximately 0.03% (Figure 6). GTDB-Tk taxonomy assigned these homologs to eight bacterial phyla, with Pseudomonadota accounting for 78 hits (75.0%). Within this phylum, *Vibrionaceae* (24 hits) and *Burkholderiaceae* (21 hits) were particularly abundant, while *Bdellovibrionota* (9), *Desulfobacterota* (8), *Bacteroidota* (3), *Cyanobacteriota* (3), *Nitrospinota* (2), *Campylobacterota* (1), and *Nitrospirota* (1) comprised the remainder, suggesting repeated cassette recruitment by these lineages. A maximum-likelihood tree constructed from these 104 mined sequences and five Chd proteins from cultivated chlorothalonil degraders (*n* = 109) placed the BJQ, CTN, XF, and TP-D1 strains within a single well-supported clade embedded in the broader Chd family. The high-identity core formed a tight cluster that spanned multiple host phyla, whereas distant homologs such as the *N. koreensis* GR20-10 *chd*-like sequence occupied outlying branches (Figure 7). Habitat analysis revealed that the 104 *chd*-encoding genomes originated from diverse environments, including marine (47, 44.8%), soil (15), freshwater (15), wetland (13), activated sludge (4), saline lake (2), deep subsurface (1), and glacier (1) habitats, with 7 unclassified (Figure 7). Although the BJQ isolates were recovered from terrestrial agrochemical-contaminated sites, this broad environmental distribution indicates that the cassette is not restricted to soil. Collectively, the recurrence of near-identical *chd* cassettes across geographically distant and ecologically distinct genomes, coupled with their wide habitat range, supports horizontal dissemination of a pre-adapted module rather than vertical descent as the primary driver of chd distribution.

**Figure 6:**
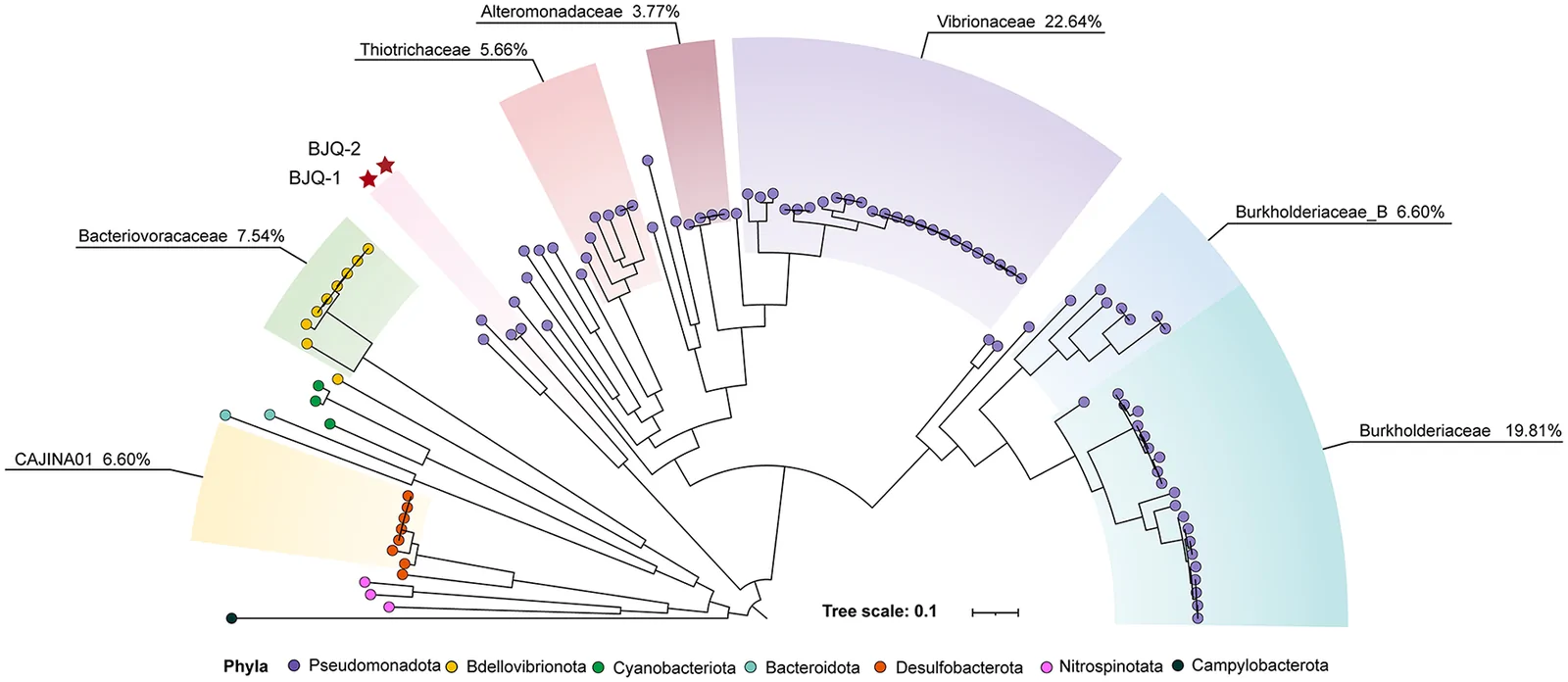
Global phylogeny of 104 Chd homologs retrieved from the Prokaryotic Genome dataset. The maximum-likelihood tree was inferred using FastTree v2 based on sequences that met the screening criteria relative to BJQ-1 Chd, including identity ≥50%, query coverage >50%, and *E*-value <1 × 10^−10^. Branches are colored by GTDB-Tk phylum, while outer arcs indicate the predominant families and their respective proportions among the 104 genomes. The newly isolated strains BJQ-1 and BJQ-2 are marked with asterisks.

**Figure 7:**
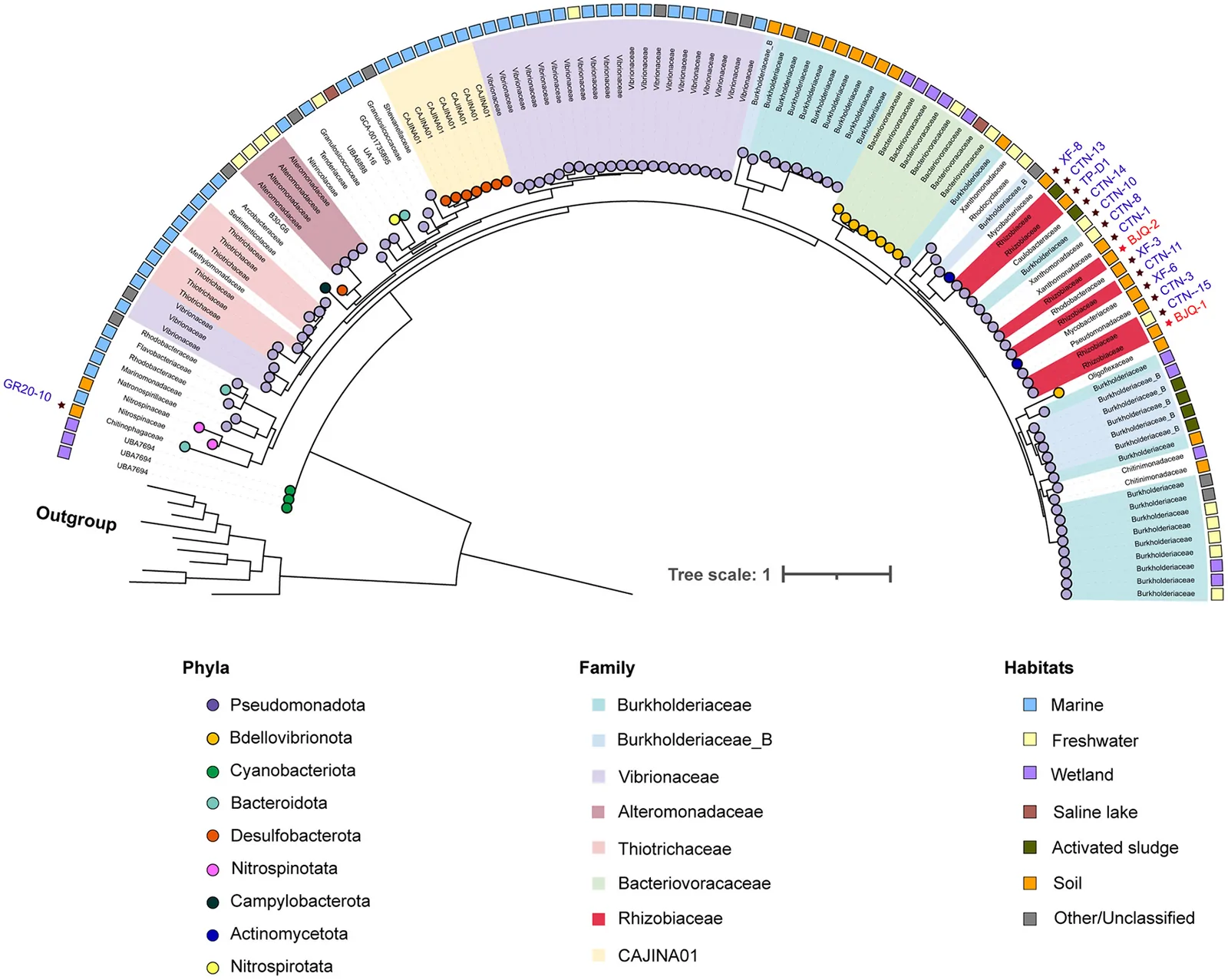
Phylogenetic distribution of 104 mined Chd homologs and five Chd proteins from cultivated chlorothalonil-degrading strains, comprising 109 sequences in total. The tree was reconstructed using the MAFFT/trimAl/IQ-TREE pipeline with 1,000 ultrafast bootstrap replicates. Tips are annotated with GTDB-Tk-derived phylum and family assignments, alongside isolation habitat. Habitats are categorized into seven types including marine, freshwater, wetland, saline lake, activated sludge, soil, and unclassified.

## 4 Discussion

The two BJQ strains were isolated from geographically separated sites yet carry a *chd* module that is essentially identical at the nucleotide level, including the upstream fusion-promoter architecture that drives its expression. Both isolates belong to the genus *Pseudochrobactrum* but represent distinct species with an ANI of 86.1% and a dDDH of 29.9%, indicating that the shared module cannot be explained by recent vertical descent from a common ancestor. The same *chd* ORF with ≥99.7% identity appears across hosts spanning several classes of Pseudomonadota (Figure 4), a pattern anticipated by Liang et al. (2011) who recovered chd from eight genera of chlorothalonil-dechlorinating strains and later supported by findings in *Pseudochrobactrum* sp. BSQ-1 (Liu et al., 2016) and RB-series isolates linking *chd* to the IS-*Olup* module (Ren et al., 2011). In our comparison the ORF is embedded in three distinct mobility configurations, namely the IS*21*-like IS-*Ocsp1* in the CTN/TP-D1 lineage, an IS*6*-family *tnpA* with a Pin recombinase in BJQ-1, and a λ Rac-like invertase in BJQ-2 (Figure 5). Across the core carriers Chd itself is 99.4–100% identical at the amino acid level whereas the flanking mobility genes are far less conserved, with *istA* and the IS6-*tnpA* of strain BJQ-1 sharing only about 55.6% identity to their CTN counterparts (Figure 5). Because the enzyme is nearly invariant while the elements that transport it differ, *chd* gene and its carriers do not share one evolutionary history. The tight cross-phylum clustering of the high-identity core in the gene-level tree argues against a single clonal sweep and favors recurrent recruitment of chd by independent mobile elements (Top and Springael, 2003; Phale et al., 2019; Bhatt et al., 2021).

The widespread dissemination of the chd cassette is facilitated not only by the mobility element but also by the upstream architecture that travels with it. Liang et al. (2011) proposed that the IRR-borne −35 box fused to the native *chd* −10 element forms a fusion promoter that drives high expression once the gene is acquired. We found that this exact motif block comprising the IRR with the embedded TTGACA −35 box, the adjacent TGGG direct repeat, and the surrounding spacer is 100% identical between BJQ-1 and CTN-1 (Supplementary Figure S6), and the conservation extends to the full 179-bp intergenic spacer in which the motif is embedded. The cassette is therefore transferred in an already active state so that the recipient gains both the enzyme and its promoter, enabling immediate expression rather than reliance on fortuitous integration near a host promoter. This pre-activation lowers the barrier for a newly acquired cassette to become functional and allows the module to behave as a plug-and-play unit in distantly related hosts, which helps explain why chlorothalonil-degrading isolates continue to emerge at contaminated sites worldwide (Liang et al., 2012; Ren et al., 2011; Yue et al., 2015; Liu et al., 2016; Top and Springael, 2003). The near-identity of the spacer between strains of different genera also constrains the timescale of dissemination and points to a recent common donor rather than ancient divergence. That the spacer is conserved together with the enzyme rather than drifting independently indicates the fusion-promoter block and chd are co-selected as a single functional unit during transfer.

Database-scale mining extends the picture from a handful of cultured strains to a global low-frequency distribution that tracks mobility rather than host phylogeny. Screening 358,092 prokaryotic genomes retrieved 104 *chd*-like sequences at a hit rate of roughly 0.03% (Figure 6). Such sparse recovery is typical of horizontally acquired elements whereas a housekeeping function would appear in a far larger fraction of host genomes (Coluzzi et al., 2022). The hits are non-randomly distributed. Pseudomonadota account for 78 of 104 sequences (75.0%) with *Vibrionaceae* (24) and *Burkholderiaceae* (21) being over-represented, a concentration that matches the pollution-exposed and metabolically versatile Pseudomonadota known to recruit mobilized catabolic pathways (Phale et al., 2019; Bhatt et al., 2021). The habitat spread is broader still and unexpected for a land-applied fungicide. Marine genomes form the largest category with 47 sequences (44.8%) ahead of soil (15), freshwater (15), and wetland (13), with smaller counts from activated sludge (4), saline lake (2), deep subsurface (1), glacier (1), and seven unclassified sources (Figure 7). Agricultural runoff and wastewater presumably carry the fungicide and its residues into coastal and freshwater systems to extend terrestrial selection pressure into the water column, or Chd may act on chlorinated aromatics beyond chlorothalonil so that the gene persists where the fungicide is absent. This latter possibility is consistent with the divergent GR20-10 *chd*-like outlier showing 44.5% identity from a ginseng-soil isolate never exposed to chlorothalonil. The taxonomic and habitat breadth of these homologs together reflect the mobility of the cassette and the widespread use of chlorothalonil rather than the phylogeny of the hosts, a pattern now documented for many mobilized catabolic pathways (Coluzzi et al., 2022; Garcillán-Barcia et al., 2025). Plasmids and transposons assemble and disseminate such pathways to allow beneficial genes to surface abruptly in distantly related hosts at polluted sites (Phale et al., 2019; Bhatt et al., 2021) (Figure 8). We acknowledge that the final count of database homologs and their habitat assignments depend on genome metadata quality which may under-represent uncultured metagenome-assembled genomes. Despite these limitations the data present chlorothalonil biodegradation as a mobilome-driven adaptation (Liang et al., 2012; Top and Springael, 2003) in which a single efficient enzyme packaged with its own promoter and mobility apparatus has disseminated across the prokaryotic tree wherever the fungicide exerts selective pressure.

**Figure 8:**
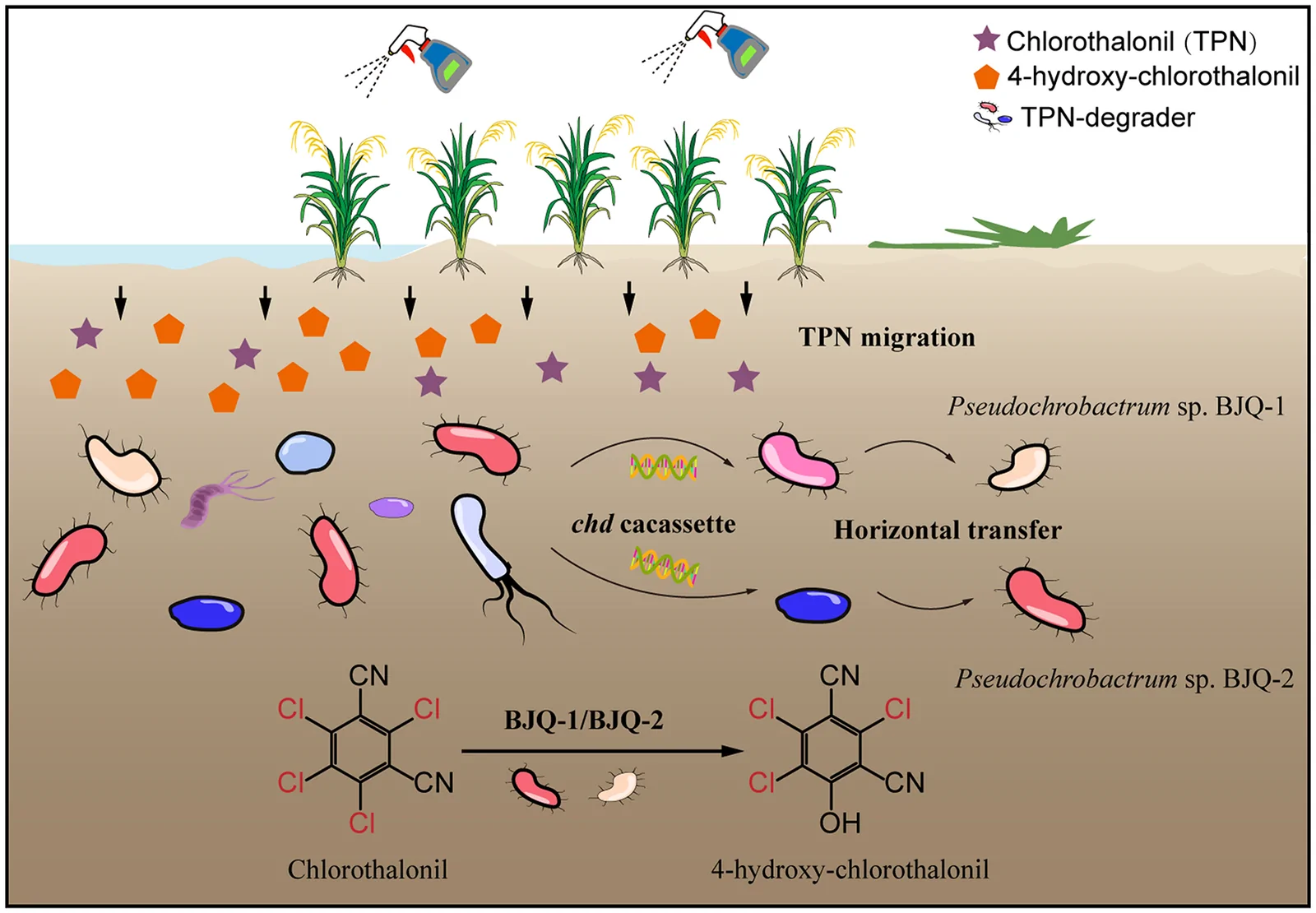
Horizontal transfer of the chd degradation gene cassette among soil bacteria.

## Data Availability

The data analyzed in this study are available in the NCBI Genbank (https://www.ncbi.nlm.nih.gov/genbank) under accession number numbers PZ225271, PZ225286, PZ786007, and PZ786008.
