# Supplementary material for: Metabolic gene analysis of newly isolated Pseudochrobactrum sp. BJQ-1 and BJQ-2 reveals global dissemination of the chlorothalonil hydrolytic dehalogenase chd gene
Source: Front Microbiol. 2026 Oct 6;17:1965943. doi: 10.3389/fmicb.2026.1965943 (PMC13682519; doi:10.3389/fmicb.2026.1965943)
Supplement: Supplementary file 1 [file Supplementary_file_1.doc]

**Supplemental Material**

**Metabolic gene analysis of newly isolated *Pseudochrobactrum* sp. BJQ-1 and BJQ-2 reveals global dissemination of the chlorothalonil hydrolytic dehalogenase *chd* gene**

Zhuang Ke^1*^, Qi Wu^2^, Min Gao^2^, Qilin Wang^3^, Lin Wang^1^, Cansheng Yuan^1*^

^1^ College of Rural Revitalization, Jiangsu Open University, Nanjing, 210036, China

^2^ Key Laboratory of Agricultural and Environmental Microbiology, Ministry of Agriculture and Rural Affairs, College of Life Sciences, Nanjing Agricultural University, Nanjing, 210095, China

^3^State Key Laboratory of Microbial Technology, School of Food Science and Pharmaceutical Engineering, Nanjing Normal University, Nanjing, Jiangsu 210000, China

^*^Address correspondence to:

Zhuang Ke, [kezhuang@jsou.edu.cn;](mailto:kezhuang@jsou.edu.cn;)
Cansheng Yuan, [yuancs@jsou.edu.cn;](mailto:yuancs@jsou.edu.cn;)

**Running title:** A mobile *chd* cassette spreads across divergent hosts

**Keywords**: chlorothalonil, hydrolytic dehalogenase, biodegradation, *Pseudochrobactrum*, horizontal gene transfer, global distribution

**Figure S1.** Hydrolysis zones of strain BJQ-1 (A) and BJQ-2 (B) on TPN-containing agar plates.

**Figure S2.** Effects of pH (A), temperature (B), and metal ions (C) on TPN removal by strains BJQ-1 and BJQ-2. Relative TPN-removal activity is shown across a pH range (3.0–10.0), a temperature gradient (15–45°C) and in the presence of eleven metal ions at 1 mM. Strain BJQ-1 and BJQ-2 are plotted separately. Data are presented as the mean ± SD of three independent experiments. Different lowercase letters above bars indicate significant differences (*P* < 0.05) among treatments as determined by one-way ANOVA followed by Tukey's HSD post-hoc test; treatments sharing the same letter are not significantly different.

**Figure S3.** Shotgun cloning and heterologous expression of the *chd* gene in *E. coli*. Clones harboring genomic inserts from strains BJQ-1 (A) and BJQ-2 (B) were selected on TPN-containing medium based on transformation efficiency. The recovered inserts encode a *chd* open reading frame (ORF) sharing ≥99.7% nucleotide sequence identity with the *chd* gene from CTN-series strains.

**Figure S4.** Heterologous expression and purification of Chd from strains BJQ-1 and BJQ-2. The *chd* genes from BJQ-1 and BJQ-2 share 100% nucleotide sequence identity. Chd was expressed in *E. coli* BL21(DE3) harboring pET-28a(+) and analyzed by SDS–PAGE. Lanes show the induced soluble fraction (single band at approximately 33 kDa) and the cobalt-affinity-purified protein.

**Figure S5.** Functional verification of heterologously expressed Chd from strains BJQ-1 and BJQ-2. (A) Visual confirmation of TPN degradation in glass test tubes. Reaction mixtures contained TPN supplemented with purified Chd from strain BJQ-1 or BJQ-2, with Tris-HCl buffer alone serving as the negative control. (B) TPN-degrading activity of purified Chd from strain BJQ-1 on TPN-containing agar plates. A clear zone around the application site indicates TPN degradation, while no clearing was observed for the Tris-HCl buffer control. (C) TPN-degrading activity of purified Chd from strain BJQ-2 on TPN-containing agar plates. The formation of a clear zone confirms enzymatic degradation of TPN, with Tris-HCl buffer as the negative control.

**Figure S6.** Comparative analysis of the chd upstream region and IS*21*-like fusion-promoter architecture across strains BJQ-1, BJQ-2, and the CTN series. The alignment displays the 179-bp *istB*–*chd* intergenic spacer plus the first 40 bp of the *chd* leader sequence, highlighting the conserved inverted repeat right (IRR) element (red dotted box) that embeds the canonical −35 promoter motif (TTGACA, red bold) and the immediately downstream 4-bp direct repeat (DR; TGGG, green underlined), which together form a composite promoter driving chd expression. Ancestral mobile genetic elements are indicated by colored annotations for *istA*/*istB* (purple) and λRac-inv/Pin-rec (blue), while the ribosome-binding site (RBS, underlined), transcription start site (TSS, orange arrow), and core promoter module are annotated in the consensus sequence at the bottom. Dashed lines connect homologous regions across strains.
